# Closed-Loop Auditory Stimulation (CLAS) During Sleep Augments Language and Discovery Learning

**DOI:** 10.3390/brainsci14111138

**Published:** 2024-11-13

**Authors:** Vincent P. Clark, Hector P. Valverde, Mason S. Briggs, Teagan Mullins, Jacqueline Ortiz, Christopher J. H. Pirrung, Olivia S. O’Keeffe, Madeline Hwang, Sidney Crowley, Marko Šarlija, Panagiotis Matsangas

**Affiliations:** 1 Psychology Clinical Neuroscience Center, Department of Psychology, University of New Mexico, Albuquerque, NM 87131, USA; 2 Department of Information Sciences, University of Zadar, 23000 Zadar, Croatia; msarlija23@unizd.hr; 3 Independent Researcher, Del Mar, CA 92014, USA

**Keywords:** electroencephalogram, EEG, sleep, learning, long-term memory, memory consolidation, neuromodulation

## Abstract

**Background/Objectives:** Slow oscillation (SO) brainwaves observed during sleep have been shown to reflect the process of memory consolidation, that underlies the critical role of sleep in learning, memory, and other cognitive functions. Closed-loop auditory stimulation (CLAS) uses tones presented in phase with SOs to increase their amplitude and number, along with other brainwave signatures related to memory consolidation. Prior studies have found that CLAS maximizes the ability to perform rote memorization tasks, although this remains controversial. The present study examined whether CLAS affects a broader range of learning tasks than has been tested previously, including a rote language learning task requiring basic memorization and also two discovery learning tasks requiring insight, hypothesis testing, and integration of experience, all processes that benefit from memory consolidation. **Methods:** Twenty-eight healthy participants performed language and discovery learning tasks before sleeping in our laboratory for three continuous nights per week over two weeks, with verum or control CLAS using a prototype NeuroGevity system (NeuroGeneces, Inc., Santa Fe, NM, USA) in a crossed, randomized, double-blind manner. **Results**: Language learning showed a 35% better word recall (*p* = 0.048), and discovery learning showed a 26% better performance (*p* < 0.001) after three continuous nights of CLAS vs. control. EEG measures showed increased SO amplitude and entrainment, SO-spindle coupling, and other features that may underlie the learning benefits of CLAS. **Conclusions:** Taken together, the present results show that CLAS can alter brain dynamics and enhance learning, especially in complex discovery learning tasks that may benefit more from memory consolidation compared with rote word pair or language learning.

## 1. Introduction

Memory consolidation is a fundamental process that processes newly acquired information and integrates it into long-term memory stores. Memory consolidation occurs during sleep, particularly during slow-wave sleep (SWS), a stage characterized by slow oscillations (SOs) in the electroencephalogram (EEG), which are large, low-frequency EEG waves in the 0.1–1 Hz range. The critical role of sleep in memory consolidation has been extensively documented in many previous studies, with disruptions in sleep adversely affecting memory retention and other cognitive function [1].

Memory consolidation during sleep is a complex process involving the reactivation and replay of memories, where neurons in the hippocampus and neocortex display the coordinated reactivation of firing patterns that were previously evoked by waking experience. This activity is coincident with sharp wave-ripples (SWRs) observed in the hippocampus, which are also synchronized with spindle activity in the neocortex [2,3]. Memory consolidation also involves the integration of new and old information [4,5] and emotional memory processing [6]. SOs originating from the neocortex act to synchronize other brain rhythms, such as thalamocortical spindles and hippocampal ripples, which are essential for the transfer of information from the hippocampus to long-term storage sites in the neocortex [7], and thus play a crucial role in coordinating the reactivation and redistribution of memory traces. These cognitive and neuronal aspects of memory consolidation are thought to contribute to the strengthening of memory traces, integration and abstraction of knowledge, emotional regulation, and creativity, among other cognitive functions.

Recent advances in cognitive neuroscience have explored various interventions to enhance memory consolidation during sleep in order to enhance these cognitive processes. We have previously used closed-loop transcranial alternating current stimulation (CL-tACS) to enhance SOs [8,9]. Small alternating electric currents were applied to the scalp at the same phase and frequency as endogenous SOs in order to increase their number and amplitude, leading to improved memory consolidation, as evidenced by improved recognition and retrieval after sleep, and also improved sleep quality [10]. The theoretical basis for CL-tACS stems from the hypothesis that by reinforcing slow oscillations through precisely timed stimulation, CL-tACS facilitates this inter-regional communication and enhances the memory consolidation process. While we and others have shown this to be efficacious, the complexity of applying current at the same phase and frequency of endogenous SOs makes this method difficult to use in practice, and is impractical to use at home independently by an end user.

Another promising approach is the use of closed-loop auditory stimulation (CLAS), which involves delivering short (~50 msec) auditory cues synchronized in time with the rising or positive phase of SOs. This method has been shown to effectively amplify SOs, thereby enhancing the consolidation of declarative memories. Several prior studies have demonstrated the efficacy of CLAS in augmenting memory performance. For instance, Ngo and colleagues [11] found that presenting short bursts of pink noise during the up-state or positive phase of SOs significantly improved word-pair retention in healthy adults.

Prior studies using CLAS have primarily used rote learning paradigms such as the Paired Associates Task, which involves learning word pairs, while more complex forms of learning have not previously been examined or have failed to produce significant memory benefits [12]. One type of learning that has not been previously attempted with CLAS is called discovery learning, where participants take the lead in the learning process and must create and test hypotheses about the best way to solve the learning task. Participants are not given any explicit clues but must learn to solve the task through the course of training by actively interacting with task stimuli, recollecting details of prior stimulus events and feedback (which indicated whether they had made a correct or incorrect response), then generating and testing hypotheses regarding the best way to solve the task.

In this paper, we aimed to investigate the effects of multi-night CLAS on memory consolidation during sleep, focusing on its potential to enhance memory performance in a number of declarative learning tasks that have not been previously attempted while using CLAS. These tasks were used to assess whether CLAS was able to benefit both real-world learning, in this case, foreign language learning, as well as whether CLAS benefits the memory distillation and consolidation processes required for discovery learning [4]. By leveraging rigorous experimental protocols, we sought to provide a comprehensive understanding of how CLAS can be optimized for cognitive enhancement, education, and therapeutic interventions.

## 2. Materials and Methods

### 2.1. Participants

Twenty-eight participants (18–40 years of age) were recruited through social media, email, online website listings, and physical bulletins and flyers. Exclusion criteria included current intoxication or illness at intake or during the study, self-reported chronic brain or mental illness including epilepsy, migraine, substance dependence or addiction, a history of head injury with loss of consciousness for over 30 min, metal implants or non-removable metal piercings that could interfere with EEG, uncorrected vision or hearing impairment (assessed by testing in the lab), a helper animal, medications, or drugs with the potential to significantly affect sleep or brain activity, sleep disorders including a regular need to get out of bed during the night, and incompatible work or other schedules. Subjects who had previously learned Japanese or Mandarin or who had previously performed the discovery learning tasks used here were also excluded. Also, a number of participants were excluded from the analysis due to missing data (did not participate in all data collection sessions) due to unanticipated personal, work, or school-related issues, illness (such as COVID-19), and technical or other problems. In total, data from 20 participants were used for analysis.

### 2.2. Procedure

**Figure 1:**
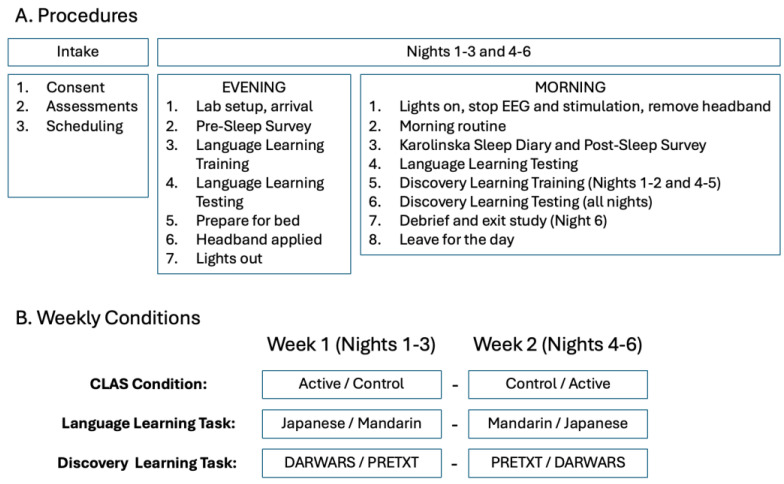
Shows sequence of procedures (**A**) and balancing of conditions across weeks (**B**).

Procedures are illustrated in Figure 1. First, eligible participants were brought into the laboratory for an intake session, which began with reading and signing a consent form. Next, participants were assessed using the Big Five Inventory 10 for personality [13] and the Shipley-2 IQ test [14]. Sleep patterns and fatigue were assessed using the Owl Lark Self-Test [15], Fatigue Severity Scale [16], Epworth Sleepiness scale [17], Athens Insomnia scale [18], Pittsburgh Sleep Quality Index [19], and Stanford Sleepiness scale [20]. All visits included the Karolinska Sleep Diary questionnaire [21] for the previous night’s sleep.

Subjects were scheduled for six overnight sessions in the lab, divided into two sets of three nights each (referred to here as weeks) with at least one night in between weeks, with each using verum or control CLAS, with condition order randomized between subjects.

For stimulation and task conditions, 10 subjects began with control, 10 with verum, 10 started with Japanese, 10 with Mandarin, and 10 started with PRETXT, 10 with DARWARS. In keeping with the crossed within-subject design, subjects received the opposite stimulation condition (e.g., verum vs. control) and opposite tasks (e.g., Mandarin vs. Japanese for the Language Learning task and DARWARS vs. PRETXT for the Discovery Learning task) on the second week.

Each night began with the experimenters setting up the sleep laboratories with fresh bedsheets, ensuring that the computers were set up with questionnaires, tasks, and an active internet connection. After arrival on the first evening, participants performed the Karolinska Sleep Diary regarding their quality of sleep for the previous night, and participants completed a Pre-sleep Survey Form on each evening that asked about caffeine, alcohol, and other drug use for that day, as well as their current level of sleepiness and general mood state for the day. Once completed, participants performed the Language Learning Training Task and then the Language Learning Test described below. Participants then got ready for bed (change clothes, wash face, brush teeth, etc.). The NeuroGevity Headband was then applied by the participants themselves, with the experimenter watching to ensure that it was applied correctly. The recording electrodes were placed on the left and right forehead at approximately 10–20 sites Fp1 and Fp2, although the exact 10–20 placement was not verified. The ground was placed in the middle of the forehead, and the reference electrode was on the left mastoid, with the experimenter ensuring the headband was correctly placed and comfortable for the participant. The EEG signal was tested, and the recording started. A wake-up time was agreed between the experimenter and participant, and the participant turned off their phone along with any other wireless devices that could interfere with the NeuroGevity system and placed them away from the bed.

The following morning, the Experimenter knocked and entered at the prearranged time, woke the participant if not already awake, stopped EEG the recording, and removed the headband. The participant performed their morning routine and then completed the Karolinska Sleep Survey, Karolinska Sleep Diary, and Post-sleep survey forms for the prior night. The Post-Sleep survey asked questions about sleep quality, whether the participant heard “clicking” from the NeuroGevity system overnight, their level of comfort, and any side effects from wearing the headband overnight. Participants then completed another Language Learning Test and performed the Discovery Learning Training Task and testing blocks for that day. Once completed, the participant left for the day, returning for two more evenings that week, which followed the same plan. For the morning after the third night, only Language and Discovery Learning testing blocks were performed, with no Discovery Learning training (as no more CLAS treatments were scheduled for the following nights that week). For the second week, the same plan was repeated with training using the other language and the other Discovery Learning Task, with task order randomized between subjects. On the final morning after testing, participants were debriefed, reimbursement was arranged, and they exited the study.

### 2.3. Language Learning Task

The Language Learning Task was selected to examine if CLAS benefits the real-world need for accelerated language learning [22], and also due to its similarity to the Paired Associates Task used in many previous CLAS and CL-tACS sleep memory consolidation studies [12]. The Language Learning Task used here was similar to [23]. The Language Learning Task was given before bed on each study night. Each Japanese or Mandarin word was paired with the equivalent English word, with one set of three nights using Japanese and the other set of three nights using Mandarin, with the order of the specific language and stimulation condition across the two weeks randomized across participants.

Participants performed two Language Learning training blocks and one test block each night before sleep, and another test block was given each morning after waking up. During each training block, 50 English/Foreign language word pairs were shown once in randomized order. Each pair lasted on the screen for 10 s. Participants were required to type the corresponding English word while the foreign word was presented on screen for 10 s. During test sessions, participants were presented with foreign words one at a time without the English equivalent word and were asked to type the recalled English equivalent word from memory. All 50 trained words were presented once in each test block in a randomized order.

Performance accuracy during testing was assessed by comparing the typed word with the correct word. Typed responses were judged by the experimenter as correct if they exactly matched the correct word or if the typed response was intended by the participant to be the correct word but differed slightly due to a typing error (e.g., typing letters slightly out of order, such as “ie” instead of “ei”, making a phonetically similar replacement such as the letter “c” for “s” or “k”, etc.). The experimenter assessing response accuracy was blind to the stimulation condition.

### 2.4. Discovery Learning Tasks

Two discovery learning tasks were used here: the DARWARS Learning Task [24] and PRETXT Learning Task [25]. One discovery learning task was trained during two mornings over the first week (after the first two nights of verum or control CLAS), and the other discovery learning task was trained during the same two mornings on the following week, with task order and stimulation condition randomized across subjects. The final test blocks occurred the morning after the third night of sleep without an additional training block. The discovery learning task training was performed in the morning after the language learning test block was completed in order to minimize the potential for interactions or disruptions between the two learning tasks and also to test whether CLAS would benefit from the consolidation of information learned many hours before sleep and stimulation. For both discovery learning tasks, participants actively engaged in exploring and problem-solving to discover new knowledge on their own. This method contrasts with direct instruction learning tasks, where information is provided, and participants are asked to memorize it by rote, such as in the Paired Associates Task used in many prior sleep memory consolidation studies [12], and also the Language Learning Task used in the current study.

#### 2.4.1. DARWARS Task

The DARWARS task has been used in a variety of previous studies from our laboratory to examine the effects of brain stimulation on learning during wakefulness [24,26,27,28] and during sleep [8,9]. This is the first study we are aware of using CLAS as a stimulation modality with any discovery learning task. Stimuli were developed from the DARWARS virtual reality environment [29]. Five-second video clips taken from the DARWARS training environment were captured for use as feedback in the task. Six hundred still images were extracted from these videos and edited to include or remove specific target objects. For each of the images containing target objects, a corresponding image was created which did not contain a hidden target object. The images were arranged in random order and were not presented to participants in matched target object/no target object pairings.

Before training on the first morning, participants were tested for their baseline ability to detect target objects using a test block with no feedback. Participants were then trained to detect the target objects, and after training, the participants were immediately tested again. Pre- and post-training test blocks consisted of 50 images that were presented without feedback. Training consisted of 11 min blocks of 60 trials, each of which included an image and appropriate audiovisual feedback. Each image was presented for 2 s with an inter-trial interval that averaged 6 s. Testing occurred immediately after training and again the next morning after sleep. On the first two mornings, participants received one baseline block, two training blocks, and one test block. On the third, final morning, participants received 4 test blocks.

On the first morning, participants were given the following instructions: “Today, you will be viewing a series of images taken from a virtual program used to train soldiers headed to the Middle East. For each image, you will be making a decision as to whether or not you think there is a threat present in the image. To begin with, you will complete a baseline measure, followed by two training sessions. The training sessions differ in that after each response you make; you will be shown a short video clip displaying the consequences of your decision. After that, you’ll be given one more test measure similar to the previous ones. Then, you will simply fill out some exit questionnaires, and you will be all done. Do you have any questions?”. After the Baseline Test was completed, training began with the following instructions: “We will now begin the training phase. Again, the training session differs in that, after each response, you will be given feedback through a short video clip displaying the consequences of your decision”.

**Figure 2:**
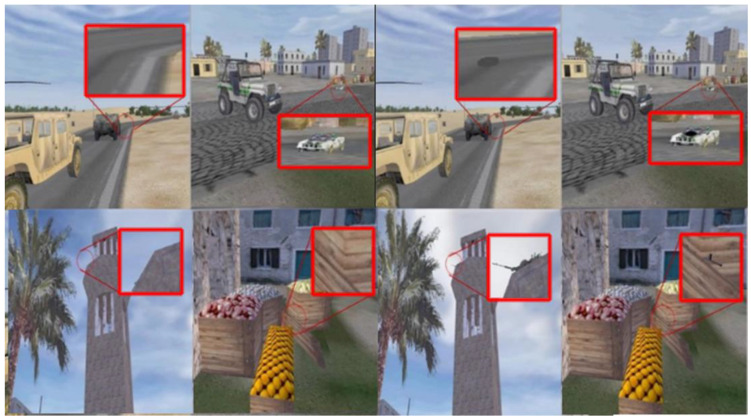
Example images presented to participants in the DARWARS task. The left of the figure contains example target-absent images and the right contains analogous target-present images. The cut-out boxes are used here for display purposes only and were not present in the actual task. The right boxes show target-present images (roadside IEDs, remote-controlled car bombs, and snipers) with the objects magnified.

Learning was accomplished by hypothesis testing and feedback. Participants were instructed to look for target threat objects and to respond as accurately as possible within two seconds when making their responses to the stimuli. No instruction is given to indicate what the target objects might look like, but it could be inferred from the response videos and memory of the image presented immediately prior. Thus, participants discovered the correct and incorrect responses to each image after receiving audiovisual feedback at the end of each training trial. Four outcomes were possible: If a concealed target object was present in the image but was missed by the subject, the feedback movie showed the outcome, e.g., a sniper attack or bomb blast occurring, which the subject could use to infer the nature of the missed target object and then detect the same or similar target object on subsequent trials. At the same time, the computer-generated voice-over indicated that the target object had been missed but gave no specific information as to the identity of the target object. If a concealed target object was present and detected, the response movie showed the scene progressing without harm, and the voiceover compliments the subject for their performance. If a concealed target object was not present, and the subject incorrectly indicated that it was present, the voice-over chastised the subject for their mistake. Finally, when there was no target present and the subject indicated this correctly, the voice-over praised the subject. See Figure 2 for example images.

#### 2.4.2. PRETXT Task

**Figure 3:**
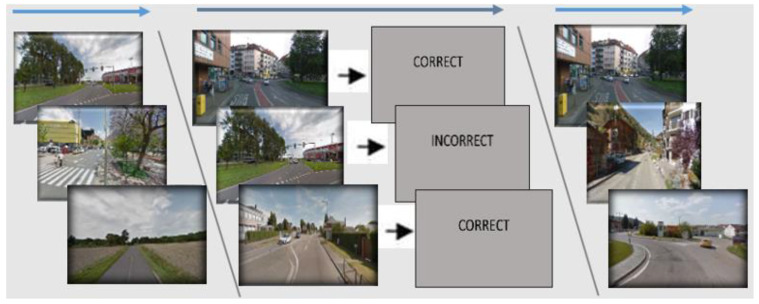
Example stimuli from the PRETXT task. The task began with a baseline test block (left column) without feedback, then a training block with feedback (middle column), and then a test block without feedback (right column).

This task was administered similar to our prior research [24] and followed a similar structure to the DARWARS Task described above. The PRETXT task focused on learning to understand and categorize pictures of European streets into two categories separated by an arbitrary rule. In order to continue using this task, the rule will not be divulged here, but the categories can be disclosed upon reasonable request to the corresponding author. Pictures were static street segment views. Each trial consisted of one street image presented for 2.5 s. Following a baseline pre-training (50 trials without feedback), each training block had 60 trials in which participants received accuracy feedback following each response. Learning was accomplished by hypothesis testing and feedback. The training was followed by another 50 trials without feedback. The baseline test took 6 min, the training portion 20 min, and the test portions 6 min. See Figure 3 for example images.

Participants were given the following instructions before the first Baseline Test Block: “Today, you will be viewing a series of pictures of European streets. For each image, you will be making a decision as to whether or not you think the picture represents category “1” or category “2”. To begin with, you will complete a baseline measure, followed by two training sessions. The training sessions differ in that after each response you make, you will receive feedback following your decision. After, you’ll be given a test measure similar to those you will have completed prior to training”. For the PRETXT Training Block, participants were given the following instructions: “We will now begin the training phase. Again, the training session differs in that, after each response, you will be given feedback through a short video clip displaying the consequences of your decision”.

### 2.5. Stimulation

During sleep, the NeuroGevity system recorded EEG using two electrodes on the forehead vs. a reference electrode placed on the left mastoid. The system analyzed ongoing EEG for the presence of SOs, and when an SO was detected on verum nights, the system presented a short (50 ms) pink noise tone pip timed to occur shortly before the positive peak of the SO. The target timing of the tone pip was 15° before the peak of the SO. The tone pips were set not to exceed 40 dB, which was strong enough to be heard but weak enough not to wake up the participant. On control nights, the EEG recording was the same, but no audible tone pips were produced. On verum nights, each initial SO stimulation was followed by a second one in real-time if a follow-on SO was detected immediately after the first SO (after the final positive-to-negative zero-crossing of the initial SO). Detection of subsequent SOs was then paused for 2.5 s, and the process repeated throughout the night.

### 2.6. EEG and Stimulus Algorithm Analysis

We assessed the neural and behavioral effects of CLAS by testing whether acoustic stimulation altered EEG signatures during sleep and whether it increased participant recall of information tested after sleep when compared with the control. Scoring of sleep stages was performed using an algorithm consistent with official guidelines [30], specifically developed for automatic sleep stage classification using prefrontal EEG data in accordance with the design of the NeuroGevity system. This eliminated the need for manual sleep stage scoring. The sleep stage classification algorithm was developed in Python and used a deep Convolutional Neural Network and Long Short-Term Memory (CNN-LSTM) network, similar to the DeepSleepNet model architecture for the sleep stage classification based on a raw single-channel EEG [31]. The model was trained on large amounts of 10 second epochs of labeled single-channel prefrontal EEG using subsets of the Montreal Archive of Sleep Studies (MASS) [32], Cleveland Family Study (CFS), Home Positive Airway Pressure (HomePAP) [33], Study of Osteoporotic Fractures (SOF) [34], and MrOS Sleep Study [35] datasets. The Fp1 and Fp2 channels were resampled from 250 Hz to 125 Hz, followed by averaging and band-pass filtering between 0.1 and 40 Hz to form a single virtual prefrontal EEG channel. The algorithm was validated on a held-out test set of multi-site PSG recordings for 5-stage classification, where each epoch is classified as either wakefulness (W) or one of the four sleep stages as defined by the American Association of Sleep Medicine (AASM) [30]: sleep stage 1 (N1—light sleep), sleep stage 2 (N2—non-REM sleep), sleep stage 3 (N3—SWS), and sleep stage REM (R). An accuracy of 84% (Cohen’s κ = 0.78) was achieved. In sleep recordings collected within the scope of this study, outputs from this sleep stage classification model were used to extract an array of basic sleep macrostructure measures. Also, information about sleep stages was used to calculate other metrics (e.g., for offline detection of SOs, for assessing the distribution of the delivered stimuli across sleep stages, etc.). In addition, a number of additional metrics were obtained from analysis and sleep scoring of the EEG recordings, including recording length (RL)—the length of the EEG record, from start in the evening to finish in the morning, total sleep time (TST)—the total time asleep based on sleep scoring, sleep-onset latency (SOL)—the time between going to bed and the first sleep time detected based on sleep scoring, and wakefulness after sleep onset (WASO)—time spent in wakefulness between first falling asleep and the final morning waking time. The results of these analyses are shown in the “Sleep Macrostructure” section of Table 1.

**Figure 4:**
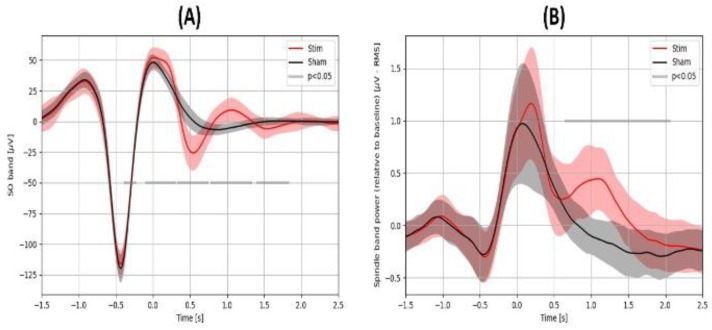
(**A**) Mean (±SD) 0.25–4 Hz filtered EEG signal, averaged across subjects, time-locked to the first auditory stimulus (t = 0 s) for the Stim and Control conditions. (**B**) Mean (±SD) of the 11–16 Hz filtered (spindle band) EEG signal amplitude envelope (based on the Hilbert transformation), averaged across subjects, time-locked to the first auditory stimulus (t = 0 s) for the Stim and Control conditions. For each stimulus, the mean spindle activity value in the 2 s period before the stimulus delivery was subtracted (which is then reflected in the y-axis values).

EEG was analyzed for a variety of characteristics [11,12,36], including (1) The proportion of second auditory stimulation, which is a measure of SO entrainment (stimulation-ratio-2-to-all). A measure of 0.5 is the highest possible value, meaning 50% of stimulations were second stimulations, i.e., each initial SO stimulation was followed by a second (follow-on) SO detection and stimulation. (2) Average peak-to-peak SO amplitude for all true positive stimuli (p2p). (3) Average absolute SO-spindle coupling for all true positive stimuli (SOSP). For each true positive stimulus, SO-spindle coupling was assessed using the time-frequency windows method introduced by McConnel and colleagues [37]. The time-frequency window was defined by the 11–16 Hz frequency range (e.g., spindle frequency range) and ±0.25 s relative to the SO positive peak. (4) Average peak-to-peak SO amplitude for initial stims only (p2p-1). (5) Average SO-spindle coupling for initial stims only (SOSP-1). (6) Average peak-to-peak SO amplitude for second stims only (p2p-2). (7) Average SO-spindle coupling for second stims only (SOSP-2). (8) Average relative SO band (0.25–4 Hz) power following a stimulus (SO-RMS-rel1). For each stimulus, the SO band signal Root-Mean-Square (RMS) value in the period of 2 s after the stimulus delivery (t = 0 in Figure 4) was divided by the SO band EEG signal RMS value in the period of 2 s before the stimulus delivery. (9) Average relative spindle band (11–16 Hz) power following a stimulus (SPN-RMS-rel1). For each stimulus, the spindle band (11–16 Hz) EEG signal RMS value in the period of 2 s after the stimulus delivery was divided by the spindle band EEG signal RMS value in the period of 2 s before the stimulus delivery. The results of these analyses are shown in the “Main EEG Variables” section of Table 1.

Analysis of the stimulation algorithm function was also performed. To detect SOs in the offline analysis, the EEG data was filtered using high-order zero-phase 0.25–4 Hz filtering. SOs detection criteria were duration criteria (corresponding to 0.5–1.5 Hz frequency range) and amplitude criteria (negative peak amplitude exceeding −80µV in negativity and peak-to-peak amplitude exceeding 120 µV) [11]. All such waveforms in the N2 and N3 sleep stages were considered offline-detected SOs. Hilbert transformation was used to compute the instantaneous phase within all offline-detected SOs. Also, a number of other stimulation algorithm performance metrics were calculated, with the target phase located at the middle of the 45–105° range, i.e., 75°. These metrics included: (1) The total number of triggered stimuli over the night (N-all-stims). (2) The total number of triggered second (follow-on) stimuli (N-2nd-Stims). (3) The total number of offline-detected SOs (N-SOs). (4) The number of true positive stimuli (stimuli falling within an offline-detected SO) and (5) The ratio between the number of true positive stimuli and the number of all triggered stimuli (Precision). (6) The ratio between the number of true positive stimuli (stimuli falling within an offline-detected SO) and the total number of offline-detected SOs (Recall). (7) The ratio between the number of stimuli falling within the target 45–105° phase range and the total number of true positive stimuli (On-time). (8) The ratio between the number of stimuli falling in the <45° phase range and the total number of true positive stimuli (Early). (9) The ratio between the number of stimuli falling in the >105° phase range and the total number of true positive stimuli (Late). (10) The mean instantaneous phases of the offline-detected SOs at the time of the stimulation for all true positive stimuli (Phase Mean). (11) The standard deviation of the instantaneous phases of the offline-detected SOs at the time of the stimulation for all true positive stimuli (Phase Std). The results of these analyses are shown in the “Stimulation Algorithm Performance Metrics” section of Table 1.

### 2.7. Behavioral Analytical Strategy

Learning scores demonstrating the effect of stimulation on consolidation in the Language Learning and Discovery Learning tasks were calculated by taking the number of correct answers obtained in the morning after sleep. We conducted a descriptive analysis of the variables of interest based on data aggregated by participant and treatment (verum vs. control nights). Second, we used linear mixed-effects model analysis to assess differences in variables of interest between the two treatment conditions. Analysis was based on two models, full and reduced. The full model included Week (first, second), Night (1 to 3) nested within Week, and treatment (verum vs. control) as the fixed effects. The participant was the random effect. The reduced model included only treatment as the fixed effect. Transformations of the dependent variables were applied to improve the statistical models’ fit. Table A1 (see Appendix A) shows the kurtosis and skewness of the model’s residual, both in their initial form and after the transformation was applied. In one case, however, the transformation was not effective, and we omitted one extreme value for the assessment of the number of nighttime stimulation events (the extreme value was over five standard deviations from the central tendency of the corresponding data). Overall, though, this shows that transformation helped to reduce kurtosis and skewness.

The next step of the analysis was focused on the effect of treatment on performance variables, i.e., variables of the Language Learning Task and Discovery Learning Tasks. The mixed effects analysis for the Language Learning Task assessed the effect of treatment on the correct number of responses in terms of English words correctly typed in response to the foreign word prompt. Language (Japanese and Mandarin), week (1 vs. 2), night (nested within a week), and treatment (verum vs. control) were the fixed effects, and Subject was the random effect, using data obtained from 19 participants, with 1 dataset lost due to technical difficulties specific to the Language Learning task. The dependent variable was the number of correct responses from the test block obtained the morning after sleep, subtracting the corresponding number of correct responses in the first evening test block (baseline).

Also, a mixed effects analysis was used to assess the effect of treatment on Discovery Learning task scores. Task (DARWARS, PRETEXT) and treatment (verum vs. control) were the fixed effects, and the participant was the random effect, using data obtained from 18 participants, with two datasets lost due to technical difficulties specific to the Discovery Learning tasks. The dependent variables were the test block scores obtained in the morning after the second night of treatment, the evening before the third night of treatment, and the morning after the third night of treatment. Test scores were adjusted by subtracting the baseline test block score obtained on the first day.

Statistical analysis was conducted with JMP statistical software (JMP Pro 17; SAS Institute; Cary, NC, USA). Data normality was assessed with the Shapiro-Wilk W test. Summary data are reported as mean ± standard deviation (M ± SD) or median—MD (interquartile range—IQR) as appropriate. An alpha level of 0.05 was used to determine statistical significance. Post-hoc statistical significance was assessed using the Benjamini–Hochberg False Discovery Rate (BH-FDR) controlling procedure with q = 0.20 [38]. Effect size analysis was conducted on data aggregated by participants and treatment based on Hedges’ g and Cohen’s d [39].

## 3. Results

Twenty subjects were tested, with 8 males, 11 females, and 1 nonbinary. The average age was 21.95 y.o. (SD 4.12). Participants were slightly above average in intelligence, with an average AQ standard score using the Shipley-2 of 111.3 (SD 12.6), Vocabulary 109.7 (SD 10.4), and Abstraction 109.0 (SD 12.1), with the population average for this test being 100. The average rating for the Karolinska Sleep Diary was 23.33 (2.0 SD), for the Athens Insomnia Scale 3.75 (2.05 SD), for the Epworth Sleepiness Scale 6.60 (3.44 SD) and Fatigue Severity Scale 31.85 (8.09 SD). All values were within the normal range, although with some evidence of mild fatigue before starting this study, as the Fatigue Severity Scale score of 31.85 is near the threshold of 36 to be considered suffering from fatigue and needing further evaluation by a physician [16].

### 3.1. Stimulation Algorithm Performance Metrics

There was a median of 682 stimulation events per participant per night. Analysis of the distribution of stimulation events by sleep stage showed that the majority (approximately 80%) occurred during N3 stage sleep, with approximately 18.5% occurring during stage N2 sleep (or 25% of the total number occurring during the N3 stage), with the remaining 1.7% occurring during other sleep stages and the waking state. There was little or no difference between verum and control CLAS nights, which were determined using the same algorithm but without any audible stimulation events being presented during control nights. The number of second stims (N-2nd-stims) was a median of 88.2 for control and 134 for verum, a 52% increase, suggesting that the algorithm detected a greater number of follow-on SOs in the verum condition due to the effects of stimulation.

### 3.2. EEG Results

A variety of EEG effects were observed in the present dataset. Significant differences were observed for all tested EEG measures, including the SO peak-to-peak amplitude (p2p), for both the initial SO (p2p-1) and subsequent SO after stimulation (p2p-2). SO RMS amplitude (SO-RMS-rel1) increased by 19.4%, and spindle amplitude (SPN-RMS-rel1) increased by 7.1% with stimulation. Spindle amplitude (SPN-RMS-rel1) showed the largest effect size (Hedges’ g of 1.65) of any EEG amplitude measure measured here. Stimulation-ratio-2-to-all (the number of second SOs that were detected immediately following the initial SO) increased by 71%, with a Hedges’ g of 1.70. Figure 4 shows the average EEG time-locked to the auditory stimulus. In general, the verum stimulus changed the SO amplitude relative to control at a number of time points in both the low bandpass SO range (Figure 4A), where it produced a longer series of approximately 1 Hz SOs, which slowly diminished in amplitude. In the spindle-frequency range (Figure 4B), a second peak of spindle frequency power can be observed at approximately 1 s. after the first peak. All of these EEG changes with stimulation suggest that the NeuroGevity system was able to evoke changes in a variety of EEG signatures related to memory consolidation.

### 3.3. Behavioral Performance Results

A mixed effects analysis was used to assess the effects of CLAS on the Language Learning Task, as assessed by the correct number of responses during testing. Language, trial (nested within language), and treatment were the fixed effects, whereas participant was the random effect (n = 18, with data from two subjects removed due to technical difficulties). The dependent variable was the transformed number of adjusted correct number responses in the last morning after subtracting the corresponding number of correct responses in the first evening baseline test. The transformation improved skewness from −0.27 to −0.05. Imputation was applied to two missing responses by their expected value. Results showed that the number of correct responses in the stim condition was higher compared to the control condition (*p* = 0.048) with an effect size of 0.374 (Hedges’ g for paired samples with data aggregated by participant and treatment). The corresponding Cohen’s d effect size is approximately 0.393. Percentage-wise analysis of data aggregated by participant and condition showed that the verum condition had a 35% higher improvement compared to the control condition.

A mixed effects analysis was used to assess the effect of treatment on Discovery Learning Task scores, with DARWARS and PRETEXT scores analyzed together. Task (DARWARS, PRETEXT) and treatment were the fixed effects, whereas participant was the random effect (n = 18, with data from two subjects removed due to technical difficulties). The dependent variable was the test score in the morning after the second night of treatment (after all training was completed), the evening before the third night of treatment, and the morning after the third night of treatment. Test scores were adjusted by subtracting the score of the first day (baseline test). Results showed that scores in the stim condition were larger compared to the control condition (*p* < 0.001), with an effect size of 0.272 (Hedge’s g for data aggregated by participant and treatment). A Hedges’ g of 0.272 corresponds to a Cohen’s d of approximately 0.286. Percentage-wise analysis of data aggregated by participant and condition showed that the stim condition had a 26% higher improvement compared to the control condition. The size of the dataset was not sufficient to examine the relationship between the EEG measures and the behavioral measures obtained.

## 4. Discussion

Memory consolidation during sleep is a multifaceted process that involves several cognitive and neuronal mechanisms. This study aimed to investigate the effects of CLAS on memory consolidation, focusing on its potential to alter EEG during sleep and to enhance declarative memory performance across a variety of learning tasks. Our findings suggest that CLAS can significantly alter EEG signatures related to memory consolidation and can improve memory consolidation, as evidenced by enhanced recall and recognition performance in the morning after sleep in both Language Learning using Japanese and Mandarin and two different Discovery Learning Tasks.

In contrast to our present findings, many prior CLAS studies of memory enhancement that have used learning tasks beyond memorization of word pairs have failed to find significant effects, even with significant changes in EEG markers of memory consolidation, such as SOs and spindles. For instance, Leminen et al. [40] compared four different memory tasks, including finger tapping, picture recognition, and face-name association tasks, but found that only a word-pair memory task showed a significant effect of CLAS. Henin et al. [41] did not find a significant effect of CLAS in a virtual reality spatial navigation task. Ong et al. [42] found no effects of CLAS on declarative memory encoding. While experimental differences in how CLAS was applied in these prior studies compared with the current study may be important, it could also be that the forms of learning examined in these prior studies did not take full advantage of the memory distillation and integration of experience offered by the memory consolidation process, which is required by discovery learning. Indeed, the hypothesis development and testing required by these learning tasks are unique, and nothing similar has been examined before using CLAS.

The cognitive aspects of memory consolidation during sleep involve the reactivation and replay of memories, synaptic homeostasis, integration of new with old information, and emotional memory processing. These processes are facilitated by neuronal activities such as SWRs in the hippocampus, which are synchronized with spindle activity in the neocortex. This coordinated reactivation during SWS is thought to help transfer memory traces from the hippocampus to the neocortex, thus stabilizing them into long-term storage [2]. Additionally, synaptic homeostasis theory posits that sleep helps maintain overall synaptic balance by downscaling less important synapses, which prevents saturation and allows for the more significant synapses to be strengthened [43,44]. This process is crucial for the integration and abstraction of knowledge, enhancement of procedural skills, emotional regulation, and creativity [2,3].

This study adds to the growing body of literature demonstrating the effectiveness of CLAS to enhance learning and memory. By delivering auditory cues synchronized with SOs during sleep, CLAS effectively amplifies these oscillations, thereby improving the consolidation of declarative memories. Previous studies have shown that CLAS can enhance paired associates learning, while other studies have failed to find other forms of learning that have benefited from CLAS [12]. The magnitude of memory enhancement from CLAS using the same DARWARS discovery learning task was similar to or slightly larger than that obtained using CL-tACS during sleep in prior studies from our laboratory [8,9]. While this prior work using CL-tACS has shown promise in laboratory settings, translating this technology to practical, at-home use has remained a challenge. The complexity of applying electrical stimulation precisely timed to the phase and frequency of endogenous SOs made this method currently impractical for independent use by end users. On the other hand, CLAS is far easier to apply, only requiring the algorithm to time the presentation of short tones relative to the large, slow oscillations that occur during memory consolidation. The technology used in the present study focused on developing a user-friendly CLAS device and protocols that could be easily implemented outside the laboratory environment in the users’ homes or other venues.

EEG data collected here are consistent with the idea that the cognitive and neuronal mechanisms underlying the behavioral enhancement using CLAS may involve the reactivation and replay of memories, synaptic homeostasis, and the synchronization of hippocampal and neocortical activity. While further research is needed to refine this technology and explore its broader effects and applications, CLAS represents a promising approach for enhancing memory consolidation and cognitive function.

### Limitations

There were a number of limitations in the current study. One was that a full dataset could only be acquired from 20 participants, with incomplete data from another 8 due to technical and other issues and data from a further 1–2 participants removed due to technical issues before each analysis was completed. While effect sizes were reasonably large for some measures, a larger sample may have allowed analysis of more details regarding the relationships between specific EEG and algorithmic measures and the amount of learning and other behavioral measures. A larger sample in future studies might also allow for analysis of the relationships between individual differences among participants, such as health, age, sleep patterns, genetics, and others, and their responses to CLAS. Also, the relatively small *p*-value obtained for the Language Learning task (*p* = 0.048) was barely below the significance threshold of *p* < 0.05 and might easily have turned out to be non-significant with slight variations in the final collected dataset. In fact, most prior CLAS studies have shown fairly small effects or none at all [12], which ultimately suggests that the conflicting results between memory consolidation studies are due to individual variability in the effects of CLAS, at least for word-pair-related tasks. Our use of 3 continuous nights of stimulation for Language Learning may have increased the effect size overall, resulting in a 35% improvement in language learning, but with a marginal level of significance. The level of significance for the Discovery Learning tasks was much larger (*p* < 0.001), showing that there may be other learning tasks aside from word-pair learning, which are more consistently sensitive across individuals to the memory consolidation benefits provided by CLAS. However, the effect size of CLAS for Discovery Learning was 27% smaller than for Language Learning, but we must also consider that only two training sessions and subsequent nights of CLAS were used for Discovery Learning, as opposed to three for Language Learning (50% more). Thus, while it’s difficult to make direct comparisons between the two types of learning tasks as used here, it does appear that even with fewer training sessions, the Discovery Learning task benefits more per night of CLAS than the Language Learning task. Future studies should examine what other types of learning tasks benefit the most from their use and also work to identify what individual differences predict the magnitude of response to CLAS, both of which may lead to more effective applications and methods of CLAS.

## 5. Conclusions

In this study, participants showed significant improvements in memory performance on both Language Learning and Discovery Learning Tasks following nights with verum CLAS compared to control conditions. Specifically, participants demonstrated better recall of Japanese and Mandarin word pairs and improved performance on the DARWARS and PRETXT discovery learning tasks. These results are consistent with previous findings while also suggesting that CLAS can facilitate the memory distillation and consolidation processes required for both language learning and more complex discovery learning tasks that require both insight and hypothesis testing. The lack of positive memory effects found in many previous CLAS studies [12] may be related to the type of learning being performed. It is possible that students and professionals who are required to learn tasks and information that involve memory distillation, hypothesis development and testing, and other cognitive functions related to the memory consolidation process might benefit most from the use of CLAS. The similarity of discovery learning to hypothesis development and required testing by many professionals, including scientists and engineers, criminal and legal experts, business entrepreneurs, medical professionals, journalists, and other professions requiring critical thinking. This suggests that CLAS may be helpful for improving performance and achieving greater success in these endeavors. However, testing in these populations will be required to confirm this.

Further studies are needed to explore the long-term effects of CLAS on memory consolidation in a wider variety of learning tasks and its potential both for real-world learning and therapeutic applications. For instance, CLAS may be a safe and effective method to enhance learning in classroom and training environments. It could also be further investigated as an intervention for individuals with dementia and other forms of memory impairment. There is also the possibility of combining CLAS with other methods that have been found to increase learning and memory, such as the application of transcranial direct current stimulation (tDCS) during training [24,25]. While tDCS applied while awake produces large effects, it lacks the potential benefits of enhanced memory consolidation during sleep offered by CLAS. Along with more intensive neuroimaging measures to assess the underlying mechanisms, analysis of participant differences that predict greater sensitivity to the learning benefits of CLAS, and improved optimization of the parameters of CLAS, together, these efforts could lead to more effective cognitive enhancement and therapeutic interventions.

## Figures and Tables

**Table 1:** Shows the aggregated values of the variables of interest by treatment (verum, control). Definitions of the variables listed are described in the methods section above. The unadjusted *p*-values derived from the two models, i.e., the full and the reduced, are shown. The full model included Week (first, second), Night (1 to 3) nested within Week, and treatment (verum vs. control) as the fixed effects. The reduced model included only treatment as the fixed effect. Results showed several interesting patterns that were consistent in both the full and the reduced models, which show large effects of CLAS on EEG during sleep.

Variable of Interest	Treatment		Unadjusted *p*-Value of Treatment		Effect Size Metric
	Control ^5^	Verum ^5^	Fixed Effects: Week, Trial (Nested), Treatment	Fixed Effects: Treatment	
Main EEG Variables					
Stimulation-ratio-2-to-all, M ± SD	0.131 ± 0.049	0.224 ± 0.055	<0.001 *	<0.001 *	1.700 ^6^
p2p, M ± SD	199 ± 18.4	206 ± 19.9	<0.001 *	<0.001 *	0.360 ^6^
SOSP, M ± SD	3.91 ± 0.936	4.11 ± 0.926	0.003 *	0.005 *	0.215 ^6^
p2p-1, M ± SD	199 ± 18.0	203 ± 19.4	0.023 *	0.021 *	0.211 ^6^
SOSP-1, MD (IQR)	3.67 (1.72)	3.89 (1.59)	0.035 *	0.047 *	0.113 ^7^
p2p-2, M ± SD	199 ± 21.1	214 ± 22.8	<0.001 *	<0.001 *	0.676 ^6^
SOSP-2, MD (IQR)	3.22 (1.62)	3.73 (1.58)	<0.001 *	<0.001 *	0.361 ^7^
SO-RMS-rel1, MD (IQR)	0.819 (0.059)	0.978 (0.090)	<0.001 *	<0.001 *	0.828 ^7^
SPN-RMS-rel1, M ± SD	1.12 ± 0.034	1.20 ± 0.053	<0.001 *	<0.001 *	1.650 ^6^
Sleep Macrostructure					
RL, M ± SD	8.19 ± 0.619	8.45 ± 0.700	0.186	0.152	0.391 ^6^
TST, M ± SD	6.79 ± 0.808	6.76 ± 1.08	0.719 ^3^	0.708 ^3^	0.031 ^6^
SOL, MD (IQR)	0.333 (0.502)	0.182 (0.514)	0.647 ^2^	0.504 ^2^	0.021 ^7^
WASO, MD (IQR)	0.562 (1.10)	0.812 (1.19)	0.409 ^2^	0.332 ^2^	0.079 ^7^
W, MD (IQR)	0.878 (1.67)	1.10 (1.25)	0.523 ^2^	0.418 ^2^	0.109 ^7^
N1, MD (IQR)	0.209 (0.206)	0.213 (0.188)	0.219 ^2^	0.150 ^2^	0.077 ^7^
N2, M ± SD	3.20 ± 0.496	3.29 ± 0.694	0.178 ^3^	0.183 ^3^	0.143 ^6^
N3, MD (IQR)	1.58 (0.700)	1.69 (0.616)	0.117	0.129	0.079 ^7^
REM, M ± SD	1.57 ± 0.497	1.55 ± 0.451	0.929	0.951	0.042 ^6^
Distribution of Stimulation by Sleep Stage					
W-stim, MD (IQR)	9.5 (13.2)	10.35 (40.5)	0.621 ^1^	0.464 ^1^	0.051 ^7^
N1-stim, MD (IQR)	0.167 (0.333)	0 (0.667)	0.571 ^4^	0.425 ^4^	0.040 ^7^
N2-stim, MD (IQR)	126 (191)	117 (254)	0.684 ^2^	0.589 ^2^	0.028 ^7^
N3-stim, MD (IQR)	544 (246)	544 (397)	0.217 ^1^	0.252 ^1^	0.036 ^7^
REM-stim, MD (IQR)	1 (5.58)	0.833 (11.9)	0.418 ^1^	0.515 ^1^	0.080 ^7^
Stimulation Algorithm Performance Metrics					
N-all-stims, MD (IQR)	731 (440)	687 (647)	0.432 ^1^	0.525 ^1^	0.041 ^7^
N-2nd-stims, MD (IQR)	88.2 (75.6)	134 (149)	<0.001 * ^1^	<0.001 * ^1^	0.327 ^7^
N-SOs, MD (IQR)	1065 (431)	1132 (792)	0.735 ^1^	0.756 ^1^	0.002 ^7^
Precision, MD (IQR)	0.774 (0.129)	0.772 (0.103)	0.768 ^3^	0.909 ^3^	0.041 ^7^
Recall, M ± SD	0.459 ± 0.089	0.443 ± 0.089	0.112 ^3^	0.152 ^3^	0.180 ^6^
Early, M ± SD	0.144 ± 0.059	0.154 ± 0.068	0.200 ^2^	0.269 ^2^	0.148 ^6^
On-time, M ± SD	0.752 ± 0.058	0.756 ± 0.067	0.775 ^3^	0.490 ^3^	0.060 ^6^
Late, M ± SD	0.104 ± 0.037	0.090 ± 0.038	0.002 *	0.002 *	0.372 ^6^
Phase mean, M ± SD	72.2 ±5.79	70.2 ± 6.23	<0.001 *	<0.001 *	0.328 ^6^
Phase std, M ± SD	31.6 ± 5.10	30.0 ± 5.25	0.022 * ^1^	0.043 *	0.309 ^6^

* Statistically significant based on the BH-FDR controlling procedure; ^1^ Data logarithmically transformed. ^2^ Data fourth root transformed. ^3^ Data square transformed. ^4^ Results were verified after excluding one outlier data point. ^5^ Descriptive statistics calculated over data generated by participants and treatment. ^6^ Hedge’s g for paired samples. Data aggregated by participant and treatment. ^7^ Non-parametric effect size metric r. Data aggregated by participant and treatment.

## Data Availability

The data presented in this study will be made available on request from the corresponding author once NeuroGeneces, Inc. receives a final response to patent applications for the NeuroGevity system.

## Appendix A

**Table A1:** Improvements in the linear mixed-effects model (LMM) residuals by applying an adjustment method (supplemental information to Table 1).

	Full LMM (Fixed Effects: Week, Trial [Nested], Treatment)					Reduced LMM (Fixed Effects: Treatment)				
Variable of Interest	Adjustment Method	Initial Model		Adjusted Model		Adjustment Method	Initial Model		Adjusted Model	
		Residuals’ Skewness	Residuals’ Kurtosis	Residuals’ Skewness	Residuals’ Kurtosis		Residuals’ Skewness	Residuals’ Kurtosis	Residuals’ Skewness	Residuals’ Kurtosis
Main variables										
Stimulation-ratio-2-to-all	None	0.64	0.90	NA	NA	None	0.64	0.77	NA	NA
p2p	None	0.73	0.31	NA	NA	None	0.76	0.42	NA	NA
SOSP	None	0.51	−0.47	NA	NA	None	0.50	−0.37	NA	NA
p2p-1	None	0.70	0.12	NA	NA	None	0.73	0.23	NA	NA
SOSP-1	None	0.50	−0.45	NA	NA	None	0.49	−0.39	NA	NA
p2p-2	None	0.57	0.03	NA	NA	None	0.58	0.16	NA	NA
SOSP-2	None	0.51	−0.39	NA	NA	None	0.52	−0.13	NA	NA
SO-RMS-rel1	None	0.69	1.71	NA	NA	None	0.70	1.92	NA	NA
SPN-RMS-rel1	None	−0.07	2.02	NA	NA	None	−0.04	1.85	NA	NA
Sleep macrostructure										
RL	None	−0.17	2.19	NA	NA	None	−0.27	2.91	NA	NA
TST	Note 3	−1.72	3.80	−0.87	0.83	Note 3	−1.95	4.69	−0.99	1.13
SOL	Note 2	3.65	19.0	−0.29	−0.58	Note 2	3.81	20.4	−0.34	−0.64
WASO	Note 2	2.42	7.36	0.87	0.19	Note 2	2.59	7.99	0.92	0.16
W	Note 2	2.37	6.74	0.81	0.26	Note 2	2.49	7.29	0.78	0.16
N1	Note 2	1.10	0.60	−0.14	−0.09	Note 2	1.22	0.86	−0.09	−0.02
N2	Note 3	−1.04	1.58	−0.20	0.10	Note 3	−1.09	1.62	−0.18	−0.12
N3	None	−0.03	0.08	NA	NA	None	−0.14	0.24	NA	NA
REM	None	−0.32	0.02	NA	NA	None	−0.32	−0.13	NA	NA
Distribution of stimulation by sleep stage										
W-stim	Note 1	3.11	14.9	0.38	−0.53	Note 1	3.82	19.7	0.50	−0.37
N1-stim	Note 4	6.45	53.2	3.04	10.9	Note 4	6.77	56.8	3.16	11.1
N2-stim	Note 2	0.83	−0.10	−0.03	−1.00	Note 2	0.83	−0.16	−0.05	−1.04
N3-stim	Note 1	1.75	2.42	−0.04	0.60	Note 1	1.74	2.37	−0.17	0.87
REM-stim	Note 1	3.18	12.5	0.99	−0.11	Note 1	3.50	15.0	1.02	−0.07
Stimulation algorithm performance metrics										
N-all-stims	Note 1	1.49	1.47	0.12	−0.11	Note 1	1.49	1.43	0.04	0.01
N-2nd-stims	Note 1	1.99	3.31	0.22	0.07	Note 1	2.00	3.40	0.18	−0.01
N-SOs	Note 1	1.64	1.97	−0.03	1.05	Note 1	1.63	1.96	−0.13	1.27
Precision	Note 3	−1.87	4.35	−1.29	2.00	Note 3	−1.96	4.61	−1.33	2.11
Recall	Note 3	−0.73	0.53	−0.22	−0.46	Note 3	−0.75	0.35	−0.21	−0.62
Early	Note 2	1.40	3.72	0.22	0.28	Note 2	1.40	3.72	0.22	0.22
On-time	Note 3	−1.10	2.10	−0.72	0.91	Note 3	−1.16	2.26	−0.77	0.99
Late	None	0.028	−0.67	NA	NA	None	0.05	−0.66	NA	NA
Phase mean	None	−0.41	−0.22	NA	NA	None	−0.40	−0.27	NA	NA
Phase std	Note 1	0.51	−0.01	0.08	−0.28	None	0.59	0.08	NA	NA

Note 1: Data logarithmically transformed. Note 2: Data fourth root transformed. Note 3: Data are squared. Note 4: Results after excluding one outlier data point.
